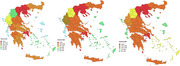# Regional and national heatmaps of dementia and mild cognitive impairment over time in Greece: Recent past and current status along with predictions for 2035 and 2050

**DOI:** 10.1002/alz.085053

**Published:** 2025-01-09

**Authors:** Konstantina Skolariki, Themis Exarchos, Vasiliki Machairaki, Constantine G. Lyketsos, Panagiotis Vlamos, Nikolaos Scarmeas, Hellenic Initiative Against Alzheimer’s Disease (HIAAD)

**Affiliations:** ^1^ Bioinformatics and Human Electrophysiology Laboratory, Ionian University, Corfu Greece; ^2^ Institute of Digital Biomedicine, Ionian University Research and Innovation Center, Corfu Greece; ^3^ Johns Hopkins University, Baltimore, MD USA; ^4^ Johns Hopkins Bayview Medical Center, Baltimore, MD USA; ^5^ School of Medicine, Johns Hopkins University, and Johns Hopkins Bayview Medical Center, Baltimore, MD USA; ^6^ Columbia University, New York, NY USA; ^7^ National and Kapodistrian University of Athens, Athens Greece

## Abstract

**Background:**

Greece has among the largest proportions of elderly worldwide and is among the fastest aging countries in Europe. Consequently, the population suffering from mild cognitive impairment (MCI) or dementia is one of the largest in Europe. This population varies significantly within the different regions in Greece.

**Method:**

We estimated the number of patients living with MCI or dementia in Greece and created heatmaps, by prefecture, for 4 different census periods, 1991, 2001, 2011 and 2021 (due to COVID results for 2021 were obtained in 2023). The prevalence of MCI and dementia, for men and women of different age categories (65‐69, 70‐74, 75‐79, 80‐84, 85+) were obtained from the *The Hellenic Longitudinal Investigation of Aging and Diet (HELIAD)*. Each prevalence for age and sex was multiplied by the respective number of people from the census, based on sex and age category.

**Result:**

The analysis showed an increase in numbers in all prefectures, ranging for MCI from 177,898 in 1991, to 223,982 in 2001, 274,872 in 2011 and 311,189 in 2021 and for dementia from 103,535 in 1991, to 113,144 in 2001, 166,774 in 2011 and 206,939 for 2021. Projected estimates for the number of people with MCI or dementia with heatmaps were generated for 2035 and 2050, based on a Greek census projection estimating the number of people above 65 and above 85, for the above years. The projection indicates a great increase in number of persons with MCI to 375,000 in 2035 and 440,000 in 2050 and for dementia to 250,000 in 2035 and 310,000 in 2050.

**Conclusion:**

Considering the fact that each person requires care over time from at least 2 other caregivers, the above numbers demonstrate the impact that the dementia pandemic will have in Greece. These heatmaps can be used as a tool in policy making, aiming to organize the setup and development of memory clinics and day care centers in the different prefectures in Greece, where the need is higher based on the number of patients.